# Automatic Aesthetics Evaluation of Robotic Dance Poses Based on Hierarchical Processing Network

**DOI:** 10.1155/2022/5827097

**Published:** 2022-09-16

**Authors:** Hua Peng, Hui Ren, Ziyang Wang, Huosheng Hu, Jing Li, Sheng Feng, Liping Zhao, Keli Hu

**Affiliations:** ^1^Department of Computer Science and Engineering, Shaoxing University, Shaoxing 312000, China; ^2^College of Information Science and Engineering, Jishou University, Jishou 416000, China; ^3^School of Computer Science and Electronic Engineering, University of Essex, Colchester CO4 3SQ, UK; ^4^Academy of Arts, Shaoxing University, Shaoxing 312000, China

## Abstract

Vision plays an important role in the aesthetic cognition of human beings. When creating dance choreography, human dancers, who always observe their own dance poses in a mirror, understand the aesthetics of those poses and aim to improve their dancing performance. In order to develop artificial intelligence, a robot should establish a similar mechanism to imitate the above human dance behaviour. Inspired by this, this paper designs a way for a robot to visually perceive its own dance poses and constructs a novel dataset of dance poses based on real NAO robots. On this basis, this paper proposes a hierarchical processing network-based approach to automatic aesthetics evaluation of robotic dance poses. The hierarchical processing network first extracts the primary visual features by using three parallel CNNs, then uses a synthesis CNN to achieve high-level association and comprehensive processing on the basis of multi-modal feature fusion, and finally makes an automatic aesthetics decision. Notably, the design of this hierarchical processing network is inspired by the research findings in neuroaesthetics. Experimental results show that our approach can achieve a high correct ratio of aesthetic evaluation at 82.3%, which is superior to the existing methods.

## 1. Introduction

Robotic dance, which combines technology and art, has become an interesting research topic [1–3]. Essentially, robotic dance is an activity of artificial intelligence that takes a robot as the carrier and dance as the content. It has attracted many researchers to work on the following aspects: cooperative human-robot dance, imitation of human dance motions, synchronization for music, and robotic choreography creation [3]. Different from other research aspects, robotic choreography creation not only involves the aesthetics of dance objects (including dance poses, dance moves, and dance works) but also requires these dance objects to conform to human aesthetics. In other words, a robot needs to master and use human aesthetic knowledge to autonomously create its dance choreography.

When a dance object involves the whole body of the robot, it can be said that the dancing object has aesthetic semantics. From the expressive dance space of humanoid robots [4], dance pose is the smallest unit with aesthetic semantics and is the basis for further constituting dance motion and robotic dance. Some researchers have explored the aesthetic problems of robotic dance poses and proposed some feasible approaches. Moreover, these approaches involve only two different categories: human subjective aesthetics [5–7] and machine learning-based methods [7–10].

To design robotic choreography, Vircikova et al. [5, 6] constructed a multi-robot system by introducing interactive evolutionary computation (IEC) and implemented a human subjective aesthetic evaluation of robotic dance poses and robotic dance motions. In [7], a theory of semi-interactive evolutionary computation (SIEC) was proposed to seek good robotic dance poses. Notably, the whole theory includes both human subjective aesthetics and machine learning-based methods: (1) in the stage of human interactive evolution, human subjective aesthetics is used for collecting aesthetic knowledge and guiding the direction of evolution; (2) in the stage of machine learning, a machine aesthetics model is trained based on the collected human aesthetic knowledge; and (3) in the stage of machine autonomous evolution, the trained machine aesthetics model independently implements aesthetics evaluation of robotic dance poses.

Furthermore, inspired by human dance behaviour, two different approaches to automatic aesthetics evaluation of robotic dance poses based on multi-modal information fusion [8, 9] were proposed. From nonvisual and visual channels, some novel features (e.g., spatial distribution feature of colour block) are extracted and fused to characterize a robotic dance pose. Based on the integrated feature, different machine learning methods are applied for training machine aesthetics models, which are further used for automatic aesthetics evaluation of robotic dance poses. In [10], a computable cognitive model of visual aesthetics was developed and applied in the automatic aesthetics evaluation of robotic dance poses. From the visual channel, three kinds of features (colour, shape, and orientation) are extracted and fused to characterize a robotic dance pose, and then machine aesthetics models are built by using different machine learning methods.

Additionally, the existing aesthetic evaluation approaches of other robotic dance objects (dance motion and dance works) still involve only two aspects: human subjective aesthetics [11–14] and machine learning-based methods [15]. In [11], a robot dance system of hip-hop was implemented, and ten people were invited to conduct an aesthetic evaluation of the generated robotic dance motions by means of questionnaire scoring. Oliveira et al. [12] implemented a real-time robot dancing framework based on multi-modal events, and empiric evaluations on robotic dance were achieved by inviting evaluators to fulfill the Likert-scaled questionnaires. Based on a cognitive architecture that integrates hidden Markov models and genetic algorithms, Augello et al. [13] implemented a computational creative agent to drive NAO robots to perform interactive dances with human partners, and audiences were invited to complete questionnaires to evaluate the aesthetics of live performances. Qin et al. [14] designed a humanoid robot dance system driven by musical structures and emotions, and subjective aesthetic evaluations on robotic dance were conducted by means of a questionnaire investigation. Moreover, we proposed a mechanism of automatic aesthetics evaluation of robotic dance motions based on multiple visual feature integration [15], and a corresponding machine aesthetics model was implemented.

However, the existing studies do not involve deep learning-based methods and ignore how to construct a brain-like intelligent system to achieve the automatic aesthetic evaluation of robotic dance poses by learning from the hierarchical visual aesthetic cognitive process of the human brain.

The main contributions of this paper are as follows:

- By learning from the visual aesthetic and cognitive mechanism of the human brain and imitating human dance behaviour, this paper explores a feasible way to develop the autonomous intelligence of robots in the field of robotic dance.
- An indirect way of visual self-perception is designed for real robots, which can make a robot observe the dance poses presented by its own whole body.
- This paper proposes a brain-like intelligent system for automatic aesthetics evaluation of robotic dance poses. In this system, a robotic dance pose is characterized by a set of features of multi-modal fusion, which integrate the features extracted in parallel by a hierarchical processing network from three different visual images (HSV, RGB, and depth). Through ordered training on each component of the hierarchical processing network, a good machine aesthetics model of robotic dance poses is constructed.
- A novel dataset of robotic dance poses, which is acquired in a real environment, is constructed and effectively verifies the approach proposed in this paper.

The rest of this paper is organized as follows: Section 2 describes the proposed methodology in detail, and Section 3 introduces the constructed dataset.  Section 4 describes the experimental environment and procedure and presents the experimental results. In Section 5, our mechanism is discussed from three aspects based on the experimental results. Finally, in Section 6, a brief conclusion and future work are given.

## 2. Methodology

### 2.1. Scene Description

A human dancer often observes the dance poses of his/her whole body through a mirror and takes them as the basis for further dance choreography after making an autonomous aesthetic evaluation. Naturally, a robot should also establish a similar mechanism to implement automatic aesthetics evaluation of its own dance poses so as to serve robotic choreography creation. This idea of developing artificial intelligence is feasible because imitating human behaviours is one of the effective learning approaches in robotics [16–18]. Considering the similarity with the human body, a biped humanoid robot is the best choice to reproduce the above human dance behaviour. For the convenience of description, the NAO robot is used as the prototype of a biped humanoid robot in this paper.

**Figure 1:**
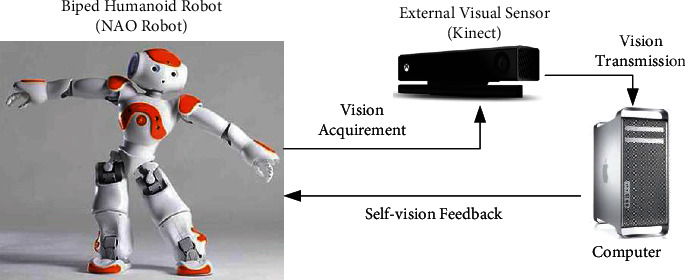
The way of indirect visual perception used in this paper.

There are two ways for a robot to observe the dance poses presented by its own whole body: direct mirror observation and indirect visual perception. In direct mirror observation, the robot is placed in front of a mirror and captures the mirror reflection of its dance poses through its own internal camera. However, when the robot performs dance poses, the perspective of its internal camera will also change frequently. In this way, the captured images of robotic dance poses will be incomplete, inaccurate, or even missing. In indirect visual perception, the robot is placed in front of an external visual sensor (e.g., Kinect) and performs its dance poses, and then the sensor is responsible for capturing these dance pose images and transmitting them back to the robot. In this way, the captured images of robotic dance poses will be complete and accurate. Therefore, this paper uses indirect visual perception as the way for a robot to perceive its own dance poses (as shown in Figure 1).

### 2.2. Framework

**Figure 2:**
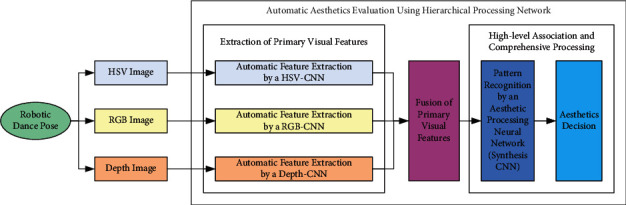
The proposed framework.

**Figure 3:**
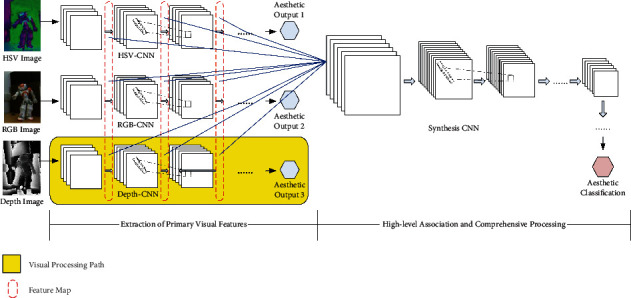
The detailed architecture of the hierarchical processing network that corresponds to the proposed framework.

Aesthetics is an advanced cognitive function of the human brain. In order to explore the aesthetic activity process and neural processing mechanism of the human brain, neuroaesthetics appears as a new research direction [19]. It comprehensively uses the methods of brain science and cognitive neuroscience to study the relationship between the human brain and aesthetic mechanism. In neuroaesthetics, Chatterjee proposed a cognitive neural model of visual aesthetics [20] and provided the following important insights: (1) The human brain processes visual aesthetics in a hierarchical way; (2) For the same visual target, multiple visual features are preliminarily processed in a group of parallel paths; (3) After multiple primary visual features are fused together, the high-level association and comprehensive processing is further carried out, and finally an aesthetics decision is made. Moreover, Wang et al. proposed an approach to image aesthetics assessment by using Deep Chatterjee's machine [21]. The above two studies inspire the framework design of this paper.  Figure 2 shows the proposed framework, and Figure 3 shows a detailed architecture of the hierarchical processing network, which corresponds to the proposed framework.

In the framework, a robotic dance pose corresponds to three kinds of images: HSV, RGB, and depth. Three parallel visual processing pathways are constructed respectively to process these three images and extract a variety of primary visual features (including edge, texture, and shape). Specifically, in each visual processing path, a convolution neural network (CNN) is constructed, and the relevant feature maps are calculated on different convolution layers. Then, the feature maps, which are generated from the above three parallel visual processing pathways, are fused together as the intermediate characterization of the robotic dance pose. Moreover, an aesthetic processing neural network (synthesis CNN) is constructed and uses the above-fused feature maps as input. After high-level association and comprehensive processing, the aesthetic processing neural network finally makes an aesthetic decision.

### 2.3. Extraction of Primary Visual Features

**Figure 4:**
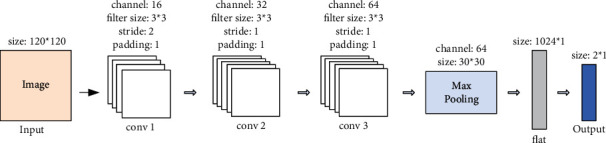
The network architecture of CNN for primary visual feature extraction.

In this stage, three parallel visual processing pathways complete the extraction of primary visual features respectively. Each visual processing pathway corresponds to a CNN, which is responsible for extracting various features (including edge, texture, and shape) from a specific type of image. Furthermore, the above features are acquired from the feature maps calculated on the convolution layers of the CNN. Notably, the same network architecture is designed for the three CNNs (HSV–CNN, RGB-CNN, and depth-CNN), as shown in Figure 4. In any of the above CNNs, all feature maps (generated by using different convolution layers) have the same size. The reason for this is to fuse the feature maps from different sources into a whole and then serve as the input of the aesthetic processing neural network.

### 2.4. High-Level Association and Comprehensive Processing

**Figure 5:**
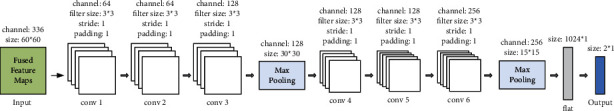
The architecture of aesthetic processing neural network.

After the stage of primary visual feature extraction, the fused feature maps form the intermediate characterization of the robotic dance pose. On this basis, an aesthetic processing neural network (a synthesis CNN) is constructed for high-level association and comprehensive processing, as shown in Figure 5. The synthesis CNN uses the fused feature maps as input, further refines the high-order visual features of robotic dance pose, and finally makes an aesthetics decision.

## 3. Dataset

**Figure 6:**
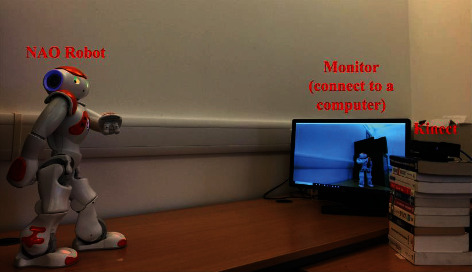
The real environment of data acquisition.

In order to verify the methodology proposed in this paper, we construct a dataset of dance poses based on real robots. Specifically, we select the NAO robot as a dance carrier and the Chinese Tibetan Tap as a dance form. Meanwhile, we use indirect visual perception as the way for the NAO robot to perceive its own dance poses (as described in Subsection 2.1). Moreover, we use Kinect as the external visual sensor that captures images of robotic dance poses. The real environment of data acquisition is shown in Figure 6.

Kinect is a powerful somatosensory device with RGB-D (RGB-depth) cameras, so we use it to directly capture RGB and depth images of robotic dance pose. Furthermore, HSV images of robotic dance poses are acquired by converting the corresponding RGB images. HSV and RGB belong to different colour spaces. To convert an image from RGB colour space to HSV colour space, the conversion details are as follows:

For a pixel of an RGB image, its colour data is assumed to be (R, G, and B). The colour data is firstly normalized to (R′, G′, and B′), and then the maximum, minimum and variation range of the three components are calculated, as shown in formulas 1–6.

(1) $$\begin{array}{c} R^{\prime }=\frac{R}{225}\text{,} \end{array}$$

(2) $$\begin{array}{c} G^{\prime }=\frac{G}{225}\text{,} \end{array}$$

(3) $$\begin{array}{c} B^{\prime }=\frac{B}{225}\text{,} \end{array}$$

(4) $$\begin{array}{c} T_{\max}=\mathrm{Max}\left(R^{\prime },G^{\prime },B^{\prime }\right)\text{,} \end{array}$$

(5) $$\begin{array}{c} T_{\min}=\mathrm{Min}\left(R^{\prime },G^{\prime },B^{\prime }\right), \end{array}$$

(6) $$\begin{array}{c} ∆=T_{\max}-T_{\min}. \end{array}$$

Next, the hue, saturation and value are calculated by using formulas (7)–(9). Finally, the pixel has its data (*H*, *S*, and *V*) in HSV colour space.

(7) $$\begin{array}{c} H=\left\{\begin{array}{cc} 0^{{}^{\circ}}\text{,} & ∆=0\text{;}\\ 60^{{}^{\circ}}\times \left(\frac{G^{\prime }-B^{\prime }}{∆}\mathrm{mod}\;6\right)\text{,} & {\;T}_{\max}=R^{\prime }\text{;}\\ 60^{{}^{\circ}}\times \left(\frac{B^{\prime }-R^{\prime }}{∆}+2\right)\text{,} & T_{\max}=G^{\prime }\text{;}\\ 60^{{}^{\circ}}\times \left(\frac{R^{\prime }-G^{\prime }}{∆}+4\right)\text{,} & T_{\max}=B^{\prime }\text{;} \end{array}\right. \end{array}$$

(8) $$\begin{array}{c} S=\left\{\begin{array}{cc} 0\text{,} & T_{\max}=0\text{;}\\ \frac{∆}{T_{\max}}\text{,} & T_{\max}\neq 0\text{;} \end{array}\right. \end{array}$$

(9) $$\begin{array}{c} V=T_{\max}\text{.} \end{array}$$

**Figure 7:**
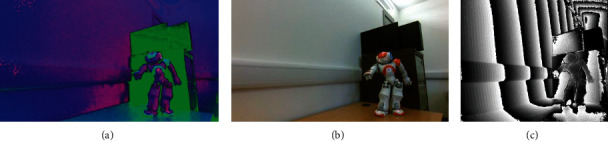
An example of a robotic dance pose. (a) HSV image. (b) RGB image. (c) Depth image.

In the whole procedure of data acquisition, 650 robotic dance poses are presented by using the NAO robot. For a robotic dance pose, three corresponding images are acquired in different ways (conversion/read from sensors): HSV, RGB, and depth (as shown in Figure 7). Thus, the whole image set of the robotic dance pose includes three image subsets: HSV, RGB, and depth, and each image subset contain 650 original images.

Notably, the aesthetic evaluation of robotic dance pose is a classification problem, so supervised learning is used to solve the problem in this paper. This requires that each robotic dance pose needs to be attached with an aesthetic label (good/bad). Moreover, considering the subjective characteristics of aesthetics, the aesthetic label provided by a human dance expert may be inaccurate. Therefore, three Chinese folk-dance experts were invited to achieve a comprehensive aesthetic annotation. Specifically, each dance expert watched the 650 robotic dance poses and then provided their corresponding aesthetic labels as good or bad. After the independent aesthetic annotations of the three dance experts, each robotic dance pose has three original aesthetic labels (good/bad). By using plurality voting on the three original aesthetic labels (good/bad), a comprehensive aesthetic label (good/bad) for a robotic dance pose is determined. Finally, the whole dataset of robotic dance poses are composed of the above image set and the corresponding comprehensive aesthetic labels.

## 4. Experiments

### 4.1. Experimental Environment

The experimental environment used in this paper includes two aspects: data acquisition and model construction. The former is used in the image acquisition process of robotic dance pose, while the latter is used to construct a model for automatic aesthetics evaluation of robotic dance pose. The details are shown in Table 1.

### 4.2. Experimental Procedure and Results

In our experiment, the whole dataset of robotic dance poses (as described in Section 3) is first divided into a training set and a test set. Moreover, the size of the training set and the test set was 80% and 20% of the dataset, respectively.

In the training stage of the hierarchical processing network, the training set is used, and the three CNNs (HSV–CNN, RGB-CNN, and depth-CNN) and the synthesis CNN are trained in turn.  Table 2 shows the training procedure of the hierarchical processing network. Notably, each robotic dance pose in the training set still corresponds to three kinds of images: HSV, RGB, and depth. Thus, the training set can still be divided into three subsets: HSV training subset, RGB training subset, and depth training subset, which are used to train the three CNNs (HSV–CNN, RGB-CNN, and depth-CNN), respectively. Moreover, each original image needs to be preprocessed before being input into the corresponding CNN for processing, including clipping, resizing, tensor conversion, and normalization. For example, the HSV, RGB, and depth images of an example robotic dance pose illustrated in Figure 3 are the results of clipping on the corresponding original images (see Figure 7).

After the training of the three CNNs is finished, three parallel visual processing pathways have been built. Thus, they can extract primary visual features from different kinds of images (HSV, RGB, and depth). For each robotic dance pose in the training set, its corresponding three images (HSV, RGB, and depth) are input into the three trained CNN models again, respectively. Then, feature maps are extracted from all the convolution layers of each CNN model, and all the feature maps, which are extracted from the three CNN models, are assembled into a set of the feature map. Next, the set of feature maps and its corresponding aesthetic label of the robotic dance pose are regarded as a training example, which is used to train the synthesis CNN. When the training of the synthesis CNN is finished, it also means that the training of the whole hierarchical processing network is completed.

In the testing stage of the hierarchical processing network, the test set is used. For each robotic dance pose in the test set, its corresponding three images (HSV, RGB, and depth) are input into the hierarchical processing network in parallel to acquire a predicted aesthetic label. By comparing the predicted aesthetic label with its corresponding real aesthetic label, it can be known whether the hierarchical processing network correctly evaluates the aesthetics of the robotic dance pose. In this way, the correct ratio of the hierarchical processing network on the test set can be calculated. The correct ratio is defined as follows:

(10) $$\begin{array}{c} Correct\;Ratio=\frac{TP+TN}{TP+TN+FP+FN}\text{,} \end{array}$$

where *TP* refers to the true positive; *FP* refers to the false positive; *TN* refers to the true negative; *FN* refers to the false egative.

Through experiments, the correct ratio of the hierarchical processing network for aesthetic evaluation is 82.3%.

### 4.3. Comparative Experiments

Based on the real dataset of robotic dance poses constructed in this paper, several comparative experiments were carried out, and some machine aesthetic models were built.  Table 3 shows the performance comparison results among these machine aesthetic models. Specifically, the comparative experiments included the following two aspects: (1) The approach in [10] was reproduced on the real dataset of robotic dance poses; (2) Corresponding to different inputs, four convolutional neural networks (CNNs) were designed and implemented respectively. The first three CNNs only use a specific type of image (HSV/RGB/depth), and there is no fusion. On the contrary, the last CNN achieves input fusion on three types of images (HSV, RGB, and depth) corresponding to a robotic dance pose. Moreover, these four CNNs all have 4 convolution layers, 2 max-pooling layers, 1 flat layer and 1 output layer.

Notably, the approaches of [8, 9] were not reproduced in the comparative experiments, but only the approach of [10] was reproduced. The reason is that the real dataset of robotic dance poses constructed in this paper is a visual dataset, and it does not contain kinematic data, while the approach of [10] only uses visual data.

Furthermore, the computation cost in Table 3 refers to the average processing time of each method for one image in the test set in seconds. Notably, the computation cost of conventional machine learning is greater than that of deep learning overall. The reason is that these methods of conventional machine learning are reproduced according to the approach proposed in [10], and a large amount of computation is spent on the extraction of hand-crafted features.

From the results of the comparative experiments in Table 3, the approach proposed in this paper achieved the best performance. We believe that this benefits from the research findings of neuroaesthetics, more specifically, from the visual aesthetic and cognitive mechanism of the human brain.

## 5. Discussion

### 5.1. Machine Aesthetics Model

Inspired by the approaches of Chatterjee [20] and Wang [21], this paper constructs a hierarchical processing network to achieve the automatic aesthetics evaluation of robotic dance poses. Essentially, the hierarchical processing network is a brain-like machine aesthetics model. Notably, it is this model that endows a robot with autonomous ability and cognitive ability so that the robot can fulfill the task of automatic aesthetics evaluation of its own dance poses.

In the dataset of robotic dance poses (as described in Section 3), each robotic dance pose corresponds to a comprehensive aesthetic label. For the comprehensive aesthetic label, three human dance experts used their own aesthetic knowledge to provide three original aesthetic labels. Then, the subjective differences in aesthetics among the three original aesthetic labels were eliminated by applying the method of comprehensive measurement, and finally, a unified value of aesthetic labels was obtained. Therefore, the dataset of robotic dance poses implies the aesthetic knowledge of human dance experts.

Furthermore, for the machine aesthetic model, there are two stages: training and testing. Essentially, the training of the machine aesthetic model aims to teach a robot to learn aesthetic knowledge, and the testing of the machine aesthetic model means that the robot uses the aesthetic knowledge it has mastered to carry out independent aesthetic evaluations. Notably, the correct ratio of the machine aesthetics model not only reflects the generalization extent of the aesthetic knowledge mastered by the robot but also reflects the consistent extent of aesthetic ability between the robot and human dance experts.

### 5.2. Versatility of the Proposed Approach

In human soaciety, there are many forms of visual art, including dance, sculpture, photography, painting, and calligraphy. As an important basis of visual arts, the aesthetic cognition based on visual understanding can drive automatic machine creations, especially robot-based automatic art creations, such as robotic choreography [4–7, 11–14], robotic photography [22], robotic painting [23], and robotic calligraphy [24].

To solve the problem of automatic aesthetics evaluation of robotic dance poses, this paper proposes a hierarchical processing network-based approach, which is inspired by neuroaesthetics. Considering the similarities in the aesthetic cognition of most visual arts, we believe that the proposed approach has certain versatility. For example, the approach can be applied to solve the problem of automatic aesthetic evaluation of sculpture. Specifically, an external visual sensor (e.g., Kinect) can be placed in front of a sculpture, and its many kinds of images (e.g., RGB, depth) will be captured. In this way, an image set of sculptures can be built. Next, human sculpture experts will be invited to make aesthetic annotations (provide aesthetic labels) after observing sculptures. Thus, the whole dataset of sculpture is composed of the above image set and the corresponding aesthetic labels. On this basis, a hierarchical processing network will be constructed and then supervised learning will be carried out. Finally, the automatic aesthetics evaluation of sculpture will be achieved. Further, once a machine possesses the abilities of visual perception and aesthetic evaluation of sculpture, it can even use a 3D printer to create sculptures autonomously.

Notably, the automatic aesthetics evaluation of robotic dance poses is a classification problem. From a deeper level, it is essentially a pattern recognition problem based on visual information. In other words, the approach proposed in this paper provides a bio-inspired solution for the pattern recognition problem. As long as the appropriate dataset is used for replacement, our approach can be easily transferred to the behaviour recognition of robots or humans. Thus, our approach can be used in multi-robot cooperation and human-robot interaction in robotics.

### 5.3. Comparison with the State-of-the-Art Approaches

Table 4 shows a comparison between the state-of-the-art approaches and our approach. Different from the existing approaches, our approach explores how to use a hierarchical processing network to achieve an automatic aesthetic evaluation of robotic dance poses.

For the highest correct ratio of the machine aesthetics model, the existing approaches achieved 81.8% [8], 81.6% [9], and 81.6% [10], respectively, and our approach achieved better performance (82.3%). We believe that this good result benefits from the research findings of neuroaesthetics, more specifically from the visual aesthetic and cognitive mechanism of the human brain. Moreover, our approach still has room to further improve the performance. We believe that the expansion of the dataset and the deepening of the hierarchical processing network will help to improve the performance.

## 6. Conclusions

This paper designs a way of indirect visual perception to enable the NAO robot to perceive its own dance poses, constructs the corresponding dataset of robotic dance poses, and presents a hierarchical processing network-based approach to automatic aesthetics evaluation of robotic dance poses. Experimental results show that a robot can visually perceive and evaluate its own dance poses, so the robot exhibits a kind of humanoid dance behaviour. This improves the autonomous ability of the robot to a certain extent and expands the research scope of artificial intelligence. Moreover, it has been proved that the brain-like intelligent system inspired by neuroaesthetics can effectively solve the problems of automatic aesthetics evaluation in the field of robotic dance. Our future work will focus on transferring and improving the proposed approach to solve the problem of automatic aesthetics evaluation of robotic dance motions.

## Figures and Tables

**Table 1:** The experimental environment in this paper.

	Hardware	Software
Data acquisition	SoftBank NAO robot V5 microsoft kinect V2 DELL PC 7080 MT	Choregraphe 2.5.10.7 kinect SDK 2.0 windows 10 visual studio 2017 opencv 3.4.5

Model construction	Sugon workstation W560-G30	Windows server 2016 PyCharm 2019.3.1 (community edition) python 3.7 pytorch 1.10.0 + cu102

**Table 2:** The training procedure of hierarchical processing network.

Input: The training set of robotic dance pose (including three subsets: HSV training subset, RGB training subset, and depth training subset), and the initial hierarchical processing network (including four initial CNNs: HSV-CNN, RGB-CNN, depth-CNN, and the synthesis CNN)
Output: The trained hierarchical processing network (including four trained CNNs: HSV-CNN, RGB-CNN, depth-CNN, and the synthesis CNN)
Steps:
1. HSV training subset is used to train HSV-CNN.
2. RGB training subset is used to train RGB-CNN.
3. Depth training subset is used to train depth-CNN.
4. Based on the above three trained CNNs (HSV–CNN, RGB-CNN, and depth-CNN), the training set of robotic dance pose (including three subsets: HSV training subset, RGB training subset, and depth training subset) is used to train the synthesis CNN.

**Table 3:** The performance comparison of different methods in comparative experiments.

Category	Method	Feature type	Type of input image	Fusion	Correct ratio (%)	Computation cost	Remarks
Conventional machine learning	Naïve Bayes	Hand-crafted features (Color + Shape + orientation)	RGB	Feature fusion	52.3	5.951	The approach in [10]
	Bayesian logistic regression				62.3	5.951	
	RBF network				53.8	5.951	
	AD tree				71.5	5.952	
	Random forest				70.8	5.951	
	Voted perceptron				71.5	5.954	
	Bagging				64.6	5.951	
	Rotation forest				70.8	5.955	
	LWL				61.5	5.992	

Deep learning	Convolution neural network	Automatically extracted features	HSV		69.2	0.020	Designed for comparison
			RGB		70.8	0.029	
			Depth		71.5	0.017	
			HSV + RGB + Depth	Input fusion	74.6	0.052	
			HSV + RGB + Depth	Feature fusion	82.3	0.103	Our approach

**Table 4:** The comparison between the state-of-the-art approaches and our approach.

	The approach in [8]	The approach in [9]	The approach in [10]	Our approach
Information channel	Nonvisual and visual	Nonvisual and visual	Visual	Visual
Cognitive model based	No	No	Yes	Yes
Feature type	Hand-crafted features	Hand-crafted features	Hand-crafted features	Automatically extracted features
Aesthetic manner	Machine learning based method	Machine learning based method	Machine learning based method	Deep learning based method
Data acquisition environment	Simulation	Simulation	Simulation	Real
Highest correct ratio	81.8%	81.6%	81.6%	82.3%

## Data Availability

The data used to support the findings of this study are available from the corresponding author upon request.

## References

[1] Aucouturier J. J. (2008). Cheek to chip: dancing robots and AI’s future. *IEEE Intelligent Systems*.

[2] Or J. (2009). Towards the development of emotional dancing humanoid robots. *International Journal of Social Robotics*.

[3] Peng H., Zhou C., Hu H., Chao F., Li J. (2015). Robotic dance in social robotics—a taxonomy. *IEEE Transactions on Human-Machine Systems*.

[4] Peng H., Li J., Hu H., Zhou C., Ding Y. (2018). Robotic choreography inspired by the method of human dance creation. *Information*.

[5] Vircikova M., Sincak P. Dance choreography design of humanoid robots using interactive evolutionary computation.

[6] Vircikova M., Sincak P. Discovering art in robotic motion: from imitation to innovation via interactive evolution.

[7] Peng H., Hu H., Chao F., Zhou C., Li J. (2016). Autonomous robotic choreography creation via semi-interactive evolutionary computation. *International Journal of Social Robotics*.

[8] Peng H., Li J., Hu H., Zhao L., Feng S., Hu K. (2019). Feature fusion based automatic aesthetics evaluation of robotic dance poses. *Robotics and Autonomous Systems*.

[9] Li J., Peng H., Hu H., Luo Z., Tang C. (2020). Multimodal information fusion for automatic aesthetics evaluation of robotic dance poses. *International Journal of Social Robotics*.

[10] Peng H., Li J., Hu H., Hu K., Tang C., Ding Y. (2020). Creating a computable cognitive model of visual aesthetics for automatic aesthetics evaluation of robotic dance poses. *Symmetry*.

[11] Shinozaki K., Iwatani A., Nakatsu R. Concept and construction of a robot dance system.

[12] Oliveira J. L., Reis L. P., Faria B. M., Gouyon F. (2012). An empiric evaluation of a real-time robot dancing framework based on multi-modal events. *TELKOMNIKA Indonesian Journal of Electrical Engineering*.

[13] Augello A., Infantino I., Manfrè A., Pilato G., Vella F., Chella A. (2016). Creation and cognition for humanoid live dancing. *Robotics and Autonomous Systems*.

[14] Qin R., Zhou C., Zhu H., Shi M., Chao F., Li N (2018). A music-driven dance system of humanoid robots. *International Journal of Humanoid Robotics*.

[15] Peng H., Hu J., Wang H. (2021). Multiple visual feature integration based automatic aesthetics evaluation of robotic dance motions. *Information*.

[16] Schaal S. (1999). Is imitation learning the route to humanoid robots?. *Trends in Cognitive Sciences*.

[17] Andry P., Gaussier P., Moga S., Banquet J., Nadel J. (2001). Learning and communication via imitation: an autonomous robot perspective. *IEEE Transactions on Systems, Man, and Cybernetics - Part A: Systems and Humans*.

[18] Breazeal C., Scassellati B. (2002). Robots that imitate humans. *Trends in Cognitive Sciences*.

[19] Zeki S., Nash J. (1999). *Inner Vision: An Exploration of Art and the Brain*.

[20] Chatterjee A. (2004). Prospects for a cognitive neuroscience of visual aesthetics. *Bulletin of Psychology and the Arts*.

[21] Wang Z., Liu D., Chang S., Florin D., Diane B., Thomas H. Image aesthetics assessment using Deep Chatterjee’s machine.

[22] Lan K., Sekiyama K. Optimal viewpoint selection based on aesthetic composition evaluation using Kullback-Leibler divergence.

[23] Greenfield G. Robot paintings evolved using simulated robots.

[24] Ma Z., Su J. (2017). Aesthetics evaluation for robotic Chinese calligraphy. *IEEE Transactions on Cognitive and Developmental Systems*.

